# A Hemin–Graphene Nanocomposite-Based Aptasensor for Ultrasensitive Colorimetric Quantification of Leukaemia Cells Using Magnetic Enrichment

**DOI:** 10.3390/bios12121070

**Published:** 2022-11-23

**Authors:** Jing Su, Liqiang Zhang, Luogen Lai, Wufu Zhu, Chong Hu

**Affiliations:** 1 School of Life Sciences, Jiangxi Science & Technology Normal University, Nanchang 330013, China; 2 School of Pharmacy, Jiangxi Science & Technology Normal University, Nanchang 330013, China

**Keywords:** graphene oxide, biosensors, hemin, sgc8c aptamer, leukaemia cells

## Abstract

Diagnostic blood cell counting is of limited use in monitoring a minimal number of leukaemia cells, warranting further research to develop more sensitive and reliable techniques to identify leukaemia cells in circulation. In this work, a hemin–graphene nanocomposite-based aptasensor was developed for ultrasensitive colorimetric detection of leukaemia cells (CEM) using magnetic enrichment. Hemin-conjugated graphene oxide nanocomposites (HGNs) were prepared by hydrazine reduction using graphene oxide nanosheets and hemins. Hence, the prepared HGNs become able to absorb single-stranded DNA and acquire peroxidase-like activity. The aptamer sgc8c, which recognizes a specific target on leukaemia cells, was absorbed onto HGNs to capture the target CEM cancer cells. The captured target cells that associated with the HGNs were then concentrated and separated by magnetic beads (MBs) coated with sgc8c aptamers, forming a HGN–cell–MB sandwich structure. These sandwich structures can be quantified via an oxidation reaction catalysed by HGNs. By utilizing dual signal amplification effects generated by magnetic enrichment and the improved peroxidase activity of HGNs, the biosensor allowed for highly sensitive detection of 10 to 10^5^ CEM cells with an ultra-low limit of detection (LOD) of 10 cells under optimal conditions. It is expected that the proposed aptasensor can be further employed in monitoring the minimal residual disease during the treatment of leukaemia.

## 1. Introduction

Leukaemia is one of the most common malignancies in the world, with over 300,000 cases diagnosed annually, causing about 281,000 deaths each year [1]. There are four major types of leukaemia, classified based on the cell type and rate of growth: acute lymphoblastic leukaemia, acute myeloid leukaemia, chronic lymphocytic leukaemia, and chronic myeloid leukaemia [2]. Acute leukaemia usually develops quickly and causes mortality within a short period, and thus requires immediate treatment [3,4]. Therefore, it has been one of the major focuses in clinical research.

The clinical significance of an early diagnosis of leukaemia for prolonging patient survival has been commonly accepted. Current diagnoses are primarily based on morphological and immunological phenotype analyses [5,6,7,8,9]. Morphological phenotyping includes the morphological observation and classification of bone marrow and blood cells, which can be complicated by the polymorphic nature of bone marrow cells and morphological alterations caused by other diseases [5,7]. Immunological diagnosis requires the identification of specific antigens on white blood cells to track the origins of the diseases and sub-classification of leukaemia through the analyses of antigens specific for individual differentiation stages [8,9]. Immunological phenotyping is advantageous with regard to accuracy; however, it is time-consuming and costly. Subsequently, flow cytometry, polymerase chain reaction, and fluorescence-based measurements have also been developed for the identification of leukaemia cells in the early diagnosis of leukaemia [6,10]. Nevertheless, these methods still suffer from sophisticated operations and require professional technicians to conduct the tests. Over past decades, with advances in nanotechnology, a number of nanomaterial-based biosensors have been developed, mainly on the basis of optical, colorimetric, and electrochemical mechanisms [11,12,13,14,15]. Due to the distinctive physical, chemical, and biological properties of diverse nanomaterials compared to bulk materials, these biosensors not only significantly improve sensitivity and accuracy in the detection of leukaemia cells but also reduce the complexity of analysis operations. The wide employment of various types of nanomaterials in the field of quantification of leukaemia cancer cells extensively promotes research on the early diagnosis of leukaemia.

Graphene-based nanomaterials are envisaged as a class of magic materials because of their unique structure and excellent mechanical, optical, and electrical properties [16,17,18]. For instance, their large surface area, covalent, and noncovalent binding capacities of biomolecules, polymers, and organic drug molecules make graphene-based nanomaterials ideal matrices for adsorption and conjugation in the fields of biosensor [19], drug delivery [20], and biomedicine [21]. For example, Asif et al. reported a Zn-NiAl layered double hydroxide/rGO superlattice-based electrochemical sensor for the simultaneous detection of dopamine, uric acid, and ascorbic acid owing to the outstanding electrocatalytic activity of the superlattice, partially because of the superb electrical conductivity of rGO [22]. Another typical example is hemin-functionalized graphene nanomaterials (HGNs), in which hemins are grafted onto the surface of graphene nanosheets via π–π bonds. HGNs inherit the strong peroxidase activity of hemin and are less prone to environmental variations [23,24]. In previous studies, graphene-based biosensors have been demonstrated to have many merits, including the composition of multiple functions, choices of detection mechanism, signal generation, and amplification, as well as easy-engineering [25,26,27,28]. Particularly, biosensors using aptamers selected through systematic evolution of ligands by exponential enrichment (SELEX) have been developed specifically against targets with sizes varying from metal ions to cells [29,30,31]. Just as in the formation of single-stranded DNA or RNA molecules, aptamers can interact with their targets in a mechanism similar to antibody–antigen association, but with improved binding capacity [32,33]. Thus, aptamer-based biosensors are advantageous for their specificity, low batch-to batch variability, stability, easy preparation, and versatile chemistry. They represent potentially new platforms for the early diagnosis of cancer.

In the present study, we combined the advantages of the specificity of aptamer sgc8c for leukaemia cells and the strong peroxidase activity of HGNs, as well as the magnetic amplification effect to develop a HGN-based aptasensor for ultrasensitive identification of leukaemia cells. In this sensing system, graphene oxide nanosheets were utilized to support hemins and sgc8c aptamers. Magnetic beads (MBs) functionalized with the sgc8c aptamer were used for the enrichment and separation of target cells from a mixture of cells. Target cells can be captured specifically by aptamers that attached on both the surfaces of GO and MBs, forming a HGN–cell–MB sandwich structure. They can then be quantified via an oxidation reaction catalysed by HGNs. The employment of graphene nanosheets for the immobilization of hemins can improve catalytic activity compared to free hemins and, thus, can amplify detection signals due to their large surface area-to-volume ratio and the enhancement of the electron transfer rate [23,24]. Furthermore, we used magnetic beads to quickly accumulate target cells that were low in abundance, which also amplified the signal. Under optimal experimental conditions, this design feature of the aptasensor significantly enhances the detection sensitivity, allowing the quantification of leukaemia cells, with numbers as low as 10 cells.

## 2. Materials and Methods

### 2.1. Reagents and Materials

Graphene oxide was purchased from XFNano Material Tech (Nanjing, China), and dimethyl sulfoxide and hemin were purchased from Sigma-Aldrich (St. Louis, MO, USA). Avidin-conjugated magnetic beads were obtained from Thermo Fisher Scientific (Waltham, MA, USA). Other chemicals were—unless otherwise specified—from Fuyu Fine Chemicals (Tianjin, China).

Oligonucleotides were synthesized and purified by Sangon Biotech (Shanghai, China). The sequences of oligonucleotides were Biotin-sgc8c: 5′-Biotin-TTTTTTTTT ATC TAA CTG CTG CGC CGC CGG GAA AAT ACT GTA CGG TTA GA-3′; DNA Probe: 5′-GAG AGA GAG AGA GAG AGA GAG AGA GAG AGA ATC TAA CTG CTG CGC CGC CGG GAA AAT ACT GTA CGG TTA GA-3′.

### 2.2. Cell Culture

A T-cell acute lymphoblast cell line CCRF-CEM (obtained from the Cell Bank of the Chinese Academy of Sciences, Shanghai, China) was maintained in a RPMI 1640 medium (Thermo Fisher Scientific, Waltham, MA, USA) supplemented with 10% (*v*/*v*) foetal bovine serum (GE Healthcare Life Science, Chicago, IL, USA), in the presence of penicillin (100 units mL^−1^, Sigma-Aldrich) and streptomycin (100 units mL^−1^, Sigma-Aldrich) at 37 °C in a humidified 5% CO_2_ atmosphere.

### 2.3. Synthesis and Characterization of Hemin-Functionalized Graphene Nanosheets

Ten milligrams of graphene oxide was dispersed into ten millilitres of water by sonication for 1 h to achieve a uniform graphene oxide solution. Then, 50 mg of hemin, 10 mL of water, 500 µL of ammonia hydroxide, and 30 µL of hydrazine hydrate were added to the system. The mixture was vortexed for 10 min before incubation at 60 °C for 4 h, which was followed by centrifugation at 16,000× *g* for 45 min. The supernatant was removed, and the pellet was washed twice, each time with 20 mL of water. The product was resuspended into 10 mL of water and stored in the dark at 4 °C. The conjugation of hemin onto the reduced graphene oxide was confirmed by atomic force microscopy (AFM, Agilent 5500), ultraviolet (UV-2450/2550, Shimadzu, Japan), and infrared (FT-IR, PerkinElmer Spectrum Two, Wellesley, MA, United States) absorption spectroscopy, as well as X-ray photoelectron spectroscopy (XPS, ESCALAB 250Xi spectrometer).

### 2.4. Preparation of DNA Probe HGNs

Fifty microlitres of HGNs (1 mg mL^−1^) was diluted with 50 µL of Tris-ethylenediaminetetraacetic acid (TE) buffer (10 mM Tris, 10 mM EDTA, pH 8.0), mixed with 100 µL of DNA probe (8 µM), sonicated for 10 min, and incubated for 24 h at 25 °C, with gentle agitation (50 rpm). Sodium chloride solution (1 M, 0.5 µL) was added every 5 h to make a final concentration of 10 mM.

The DNA probe-modified HGNs (DNA-HGNs) were collected by centrifugation at 15,000 rpm for 30 min and washed twice with 200 µL of TE buffer. The DNA-HGN product was resuspended in 200 µL of TE buffer and stored in the dark at 4 °C. The conjugation of DNA probe was confirmed by Shimadzu UV-2450/2550 spectroscopy.

### 2.5. Generation of Magnetic Capture Probe

Dynabeads M-280 Streptavidin (250 µL, 10 mg mL^−1^, Thermo Fisher Scientific, Waltham, MA, USA) were washed 3 times with 1X Bind & Washing buffer (10 mM Tris, 1 mM EDTA, 2 M NaCl, pH 7.5) and mixed with 500 µL of Biotin-sgc8c probe (500 nM) diluted with 1X Bind & Washing buffer. It was then incubated at room temperature for 20 min with agitation every 5 min. After magnetic enrichment, the supernatant was analysed by UV-vis spectroscopy; the pellet was washed twice with 500 µL 1X Bind & Washing buffer, resuspended in 500 µL 1X Bind & Washing buffer, and stored at 4 °C for further use.

### 2.6. Detection of CEM Cells

Magnetic capture probes (2.5 µL) were washed twice with 1X washing buffer (10 mM PBS, 5 mM MgCl_2_, 4.5 g L^−1^ glucose, pH 7.4) containing 1% bovine serum albumin and resuspended in the same buffer. CEM cells were then washed with 500 µL 1X washing buffer twice and resuspended in appropriate volumes of 1X washing buffer. Appropriate numbers of CEM cells in 20 µL were mixed with 2.5 µL magnetic capture probes and 50 µL DNA-HGNs and incubated at 37 °C in the dark for 30 min, with gentle agitation every 5 min. After magnetic enrichment, the pellets were washed twice with 100 µL of 1X washing buffer, before incubation with 100 µL of 3,3′,5,5′-tetramethylbenzidine (TMB, 0.5 mM) and 1 µL of 500 mM H_2_O_2_ at 37 °C in the dark for 30 min. The absorbance intensity was measured with UV-vis spectroscopy.

## 3. Results and Discussion

### 3.1. Principle of the HGN-Based Aptasensor for the Detection of Leukaemia Cells

**Figure 1:**
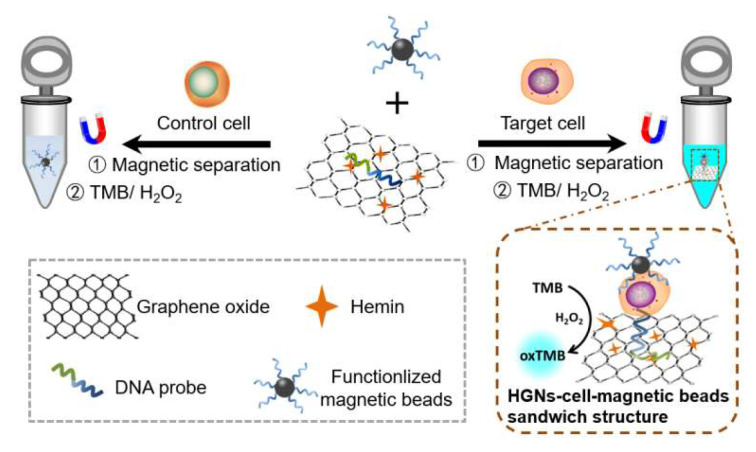
Schematic illustration of the principle for the detection of leukaemia cells based on hemin–graphene nanocomposites (HGNs) and magnetic enrichment.

The HGN-based aptasensor developed in this study combined the specificity of the sgc8c aptamer for the leukaemia cells and the strong peroxidase activity of the hemin-graphene nanocomposites (HGNs) to sensitively quantify the target cancer cells. The detection scheme is presented in Figure 1. DNA probes containing the sgc8c aptamer sequence can be adsorbed onto the surface of HGNs via π–π stacking interactions. The avidin-conjugated magnetic beads are functionalized with biotin-sgc8c aptamers to concentrate and purify the target cells from liquid matrices. In the presence of the target cells, which are the CEM cancer cells in this study, the aptamer-functionalized magnetic beads and the DNA probes on HGNs can capture and interact with the target cells to form a HGN–cell–MB sandwich structure owing to the specificity of the sgc8c aptamer. After magnetic enrichment and separation, the number of cells can be further quantified by measuring the peroxidase-mimicking activity of the HGNs. In comparison, HGNs will be removed after magnetic separation in the absence of target cells. The enriched HGNs will catalyse the oxidation and colourification of substrate 3,3′,5,5′-tetramethylbenzidine (TMB) with a maximum absorption at 650 nm in the presence of H_2_O_2_ [34]. The absorbance intensity is proportional to the number of HGN–cell–MB sandwich structures purified by magnetic separation. The biosensor developed in this paper was based on hemin-functionalized graphene oxide, upon which the DNA probes were then conjugated. The efficient grafting of hemins onto graphene oxide was confirmed by Transmission Electron Microscopy (TEM), Atomic Force Microscopy (AFM), infrared spectroscopy, and X-ray photoelectron spectroscopy. The conjugation of DNA probes to HGNs was further proved by UV spectroscopy. These quality-controlled steps, together with the highly specific affinity between the sgc8c aptamer and CEM cancer cells, ensured the efficient capture and separation of leukaemia cells with magnetic enrichment, allowing for ultrasensitive detection capability. More importantly, the sensing capacity of the biosensor can be extensively expanded when substituting the aptamer to suit to corresponding targets.

### 3.2. Preparation and Characterization of HGNs

Recent interest on graphene nanomaterials, particularly reduced graphene oxide, is largely attributed to its remarkable physicochemical features. Such features include a large surface area, high charge capacity, outstanding thermal conductivity, mechanical properties, and electron transfer capacities [19,20,21]. Modifications of graphene with appropriate functional groups confer new characteristics onto this material, which vastly expand its application in fields including biosensing, drug delivery, and biomedical science [29,31]. In our study, HGNs have been synthesized by the conjugation of GO with hemins, which generates nanocomposites with improved peroxidase-like activity compared to free hemins [23]. This allows convenient and sensitive quantification of the target, leukaemia cells, via a peroxidase catalytic activity assay. The results from this study suggest the prospective application of graphene nanomaterials in biomedicine and clinical biochemistry.

**Figure 2:**
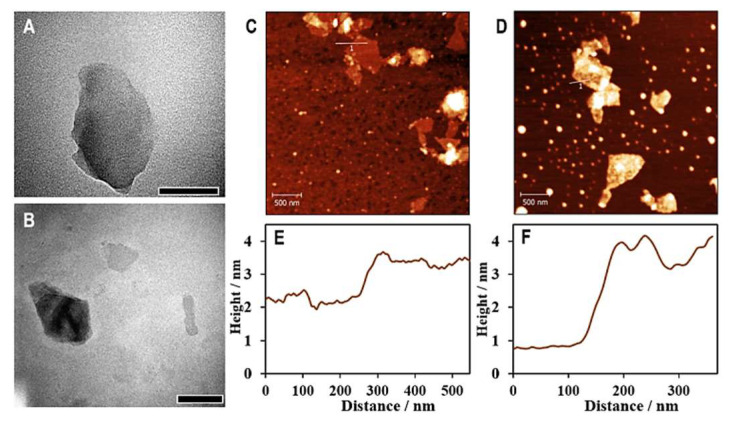
TEM and AFM analysis of GO and HGNs. TEM images of graphene oxide nanosheets (**A**) and hemin–graphene nanocomposites (HGNs) (**B**), scale bars are 500 nm long. AFM images of graphene oxide (**C**) and HGNs (**D**) show detailed surface topographies of the nanosheets before and after conjugation with hemin, respectively; cross-sectional analyses identified by the lines as graphs in AFM images shows the heights of GO (**E**) and HGNs (**F**).

To construct the aptasensor, hemin–graphene nanocomposites (HGNs) were first prepared according to previously documented with modifications [24]. The as-prepared HGNs exhibit properties of both hemins and graphene oxide. The properties are peroxidase-like activity and the ability of adsorption of single-stranded DNA (aptamers), respectively. The grafting of hemin onto graphene oxide nanosheets was confirmed by a series of instrumental analyses. The TEM and AFM images presented in Figure 2 show uniform distributions of graphene oxide and HGNs, with apparent nanosheet structures. Cross-section analyses based on AFM characterization, as plotted in Figure 2E,F, demonstrated average thicknesses of 1.1 nm and 2.6 nm for graphene oxide and HGNs, respectively, suggesting increased thickness after conjugation with hemins.

**Figure 3:**
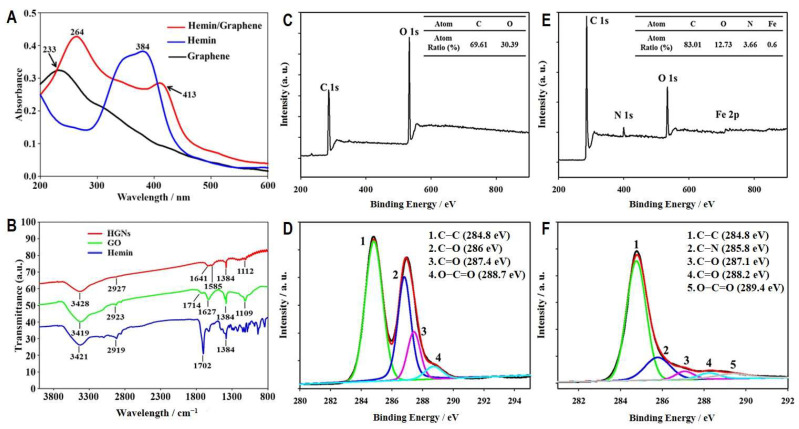
Characterization and analyses of HGNs to assess the conjugation of hemin onto the reduced graphene oxide. UV (**A**) and IR (**B**) absorption spectra of HGNs are compared with those of hemin and graphene oxide. Surface scanning patterns of graphene oxide and HGNs by X-ray photoelectron spectroscopy are shown in (**C**) and (**E**), respectively, and their carbon spectra are shown in (**D**) and (**F**), respectively.

UV spectroscopy of HGNs demonstrated characteristic absorption peaks at 264 nm and 413 nm in comparison with the characteristic peak at 233 nm for graphene oxide and 384 nm for hemin (Figure 3A), exhibiting apparent alterations in the UV spectrum caused by conjugation with hemin. In addition, as shown in Figure 3B, under infrared spectroscopy, the graphene oxide sample exhibited characteristic -OH stretching vibration at 3419 cm^−1^, C-H stretching at 2923 cm^−1^, C=C stretching at 1627 cm^−1^, C=O stretching at 1714 cm^−1^, -OH deformation at 1384 cm^−1^, and C-O-C stretching at 1109 cm^−1^. After the conjugation of hemin, shifts in the stretching vibration peaks of the oxygen bonds were observed (Figure 3B).

Additionally, we used X-ray photoelectron spectroscopy (XPS) to assess the exchange of anions on the graphene oxide surface. Compared to the survey spectrum of graphene oxide (Figure 3C), which contained 69.61% and 30.39% of carbon (C 1s) and oxygen (O 1s), respectively, HGNs showed extra peaks of nitrogen (N 1s) and ferrous (Fe 2p) with an increased carbon ratio to 83.01%, whereas the oxygen ratio decreased to 12.73% (Figure 3E). Consistent with XPS results, comparing the carbon spectra of graphene oxide (Figure 3D) and HGNs (Figure 3F) indicated significant reductions of C-O, C=O, and O-C-O after conjugation with hemins, suggesting that a reductive process occurred in graphene oxide during the grafting of hemins. The results of these analyses demonstrate that hemins were successfully conjugated onto graphene oxide nanosheets, forming HGNs.

### 3.3. Optimization of Experimental Conditions for the Aptasensor

**Figure 4:**
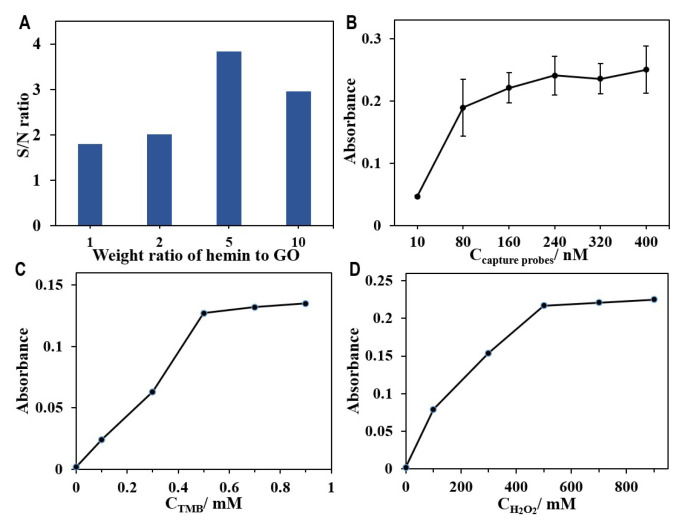
Optimization of the experimental conditions. (**A**) The signal-to-background ratios (S/N) of the aptasensor for detection of the same number of CEM cells with HGNs was prepared by varying the hemin-to-GO weight ratio from 1 to 10. (**B**) Optimization of the concentration of capture probes for preparing functionalized magnetic beads to determine CEM cells. Dependence of the concentration of TMB (**C**,**D**) H_2_O_2_ on the peroxidase-like activity of HGNs with the hemin-to-GO weight ratio of 5.

Based on the principle of the HGN-based aptasensor for the detection of leukaemia cells, the sensitivity of the biosensor significantly relates to the peroxidase activity of HGNs, which is mainly determined by the amount of hemin grafted on the nanosheets. Firstly, we optimized the synthesis parameter of HGNs with respect to the content of hemins. We prepared the HGNs by varying the hemin-to-GO weight ratio from 1 to 10. The grafting of hemin was monitored by UV spectroscopy. When increasing the amount of hemin used for HGNs preparation, a gradual increase in the characteristic absorption peak of hemin at 386 nm was observed (Figure S2A). The peroxidase activity of HGNs produced using different ratios of hemin to GO was also determined by an oxidation assay using ABTS as a substrate. With the increase in the weight ratio, more substrates can be oxidized, as shown by the intensification of the peak at 425 nm on UV spectrum (Figure S2B), indicating higher peroxidase activity of HGNs. However, while a higher input of hemin during synthesis of HGNs was favourable for the grafting of hemin onto graphene oxide, it also led to a significant increase in the background of the assay and reduced the signal-to-background (S/N) ratio. Therefore, we compared the detection capacities of HGNs produced with various ratios between hemin and GO for the quantification of a fixed concentration of CEM cells (10^5^ cells mL^−1^). The S/N ratios of assays using HGNs with hemin/graphene oxide ratios of 1, 2, 5, and 10 were investigated, and the results are presented in Figure 4A. To achieve a higher sensitivity for the following detection of CEM cells, the hemin-to-GO weight ratio of 5 was chosen for further experiments.

Besides the effect of the amount of hemin, the detection sensitivity of the aptasensor is also closely related to the concentration of capture probes used for magnetic enrichment. We optimized the concentration of capture probes when preparing the magnetic capture probes. Different concentrations of the capture probes, ranging from 10 to 400 nM, were used in the assay to determine the optimal amount of the magnetic capture probes needed (250 μL Dynabeads M-280 Streptavidin, 10 mg mL^−1^). The UV absorbance was presented in a graph against the concentration of capture probes for the detection of CEM cells at 10^5^ cells mL^−1^. Initially, the plot showed a sharp increase in the signal with the input of probes but gradually plateaued after a probe concentration of 240 nM (in Figure 3B). Therefore, the optimal concentration of the capture probes for the modification of MBs was determined to be 240 nM. The modification ratio was calculated to be 64.7% according to the absorbance before and after modification of DNA probes (Figure S3).

In addition to the amount of hemin and the concentration of magnetic capture probes, the performance of the aptasensor was also highly related to the concentration of TMB and H_2_O_2_. This directly affected the catalytic oxidation reaction of the HGNs. We repeated the previous experiment by adjusting the concentration of TMB and H_2_O_2_ to determine the optimal operating conditions. As shown in Figure 4C,D, the absorbance intensity increased with the concentrations of TMB and H_2_O_2_, and reached plateaus around 0.5 mM and 500 mM, respectively. Therefore, the concentration of TMB was fixed at 0.5 mM, and 500 mM of H_2_O_2_ was chosen for the catalytic reaction in the following experiments.

### 3.4. HGN-Based Aptasensor for Ultrasensitive Detection of CEM Cells

**Figure 5:**
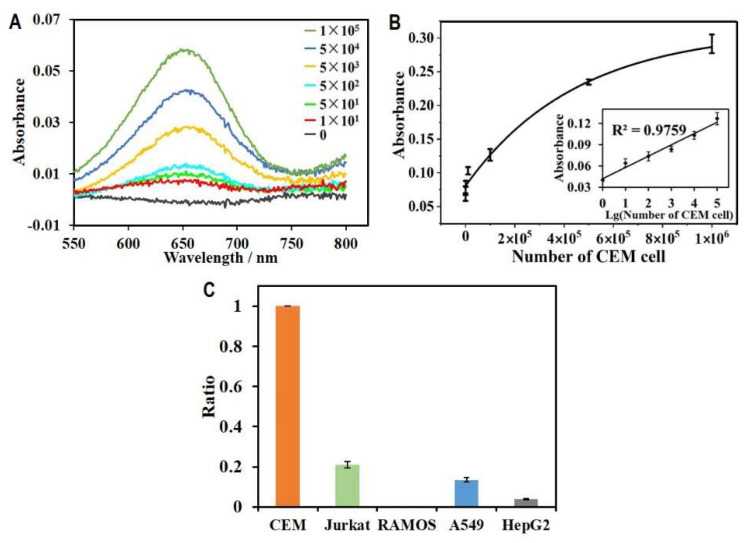
Application of the HGN-based aptasensor for quantification of CEM cells. (**A**) The UV-vis absorption spectra recorded by the HGN-based aptasensors for detecting various concentrations of target CEM cells with cell amount from 0 to 10^5^. (**B**) The relationship between absorbance intensity and the cell concentration. The inset shows the linear calibration curve between absorbance intensity and the logarithm of cell number. (**C**) Absorbance intensity ratio of control cells (including Jurkat, RAMOS, A549 and HepG2 cells) relative to the target CEM cells determined by the aptasensor.

Under optimal conditions, we applied HGN-based aptasensors for the detection of CEM cells to determine the limit of detection for the aptasensor. We prepared a series of samples that contained a range of 0 to 100,000 CEM cells. The as-prepared samples were mixed and incubated with magnetic capture probes and DNA probes HGNs for 0.5 h at 37 °C in the dark. After magnetic separation and enrichment, the mixture was then incubated with 100 µL of TMB (0.5 mM) and 1 µL of H_2_O_2_ (500 mM) for the determination and quantification of CEM cells. The UV-vis spectra corresponding to various concentration of CEM cells were recorded and overlayed in Figure 5A. Compared to the control with no cells, a distinguishable peak could be identified with as few as 10 cells. Furthermore, the increase in absorbance intensity correlated with the cell concentration in the samples, suggesting the quantification capacity of this assay for leukaemia cells in liquid samples. The calibration curve for the quantification of CEM cells is shown in Figure 4B. The absorbance intensity increased linearly with the increasing base-ten logarithm of the CEM cell number at a range of 0 to 1 × 10^5^ cells. The regression equation was Y = 0.016X + 0.0419, where Y was the absorbance intensity and X was the amount of CEM cell, and the detection limit was as low as 10 cells on the basis of the detectable signal acquired by UV-vis spectroscopy. These results indicated that our aptasensor based on sgc8c aptamers and HGNs, as well as the implemented magnetic amplification strategy, provided a highly sensitive way to concentrate and quantify CEM cells. Compared with previous biosensors reported for detection of CEM cancer cells, as summarized in Table 1, our aptasensor has superior performance in terms of detection sensitivity.

### 3.5. Specificity Assay of the HGN-Based Aptasensor

The selection of target-specific aptamers is also key for the development of an HGN-based aptasensor because the specificity of the sensor contributes to the high affinity between aptamers and their targets. The sgc8c aptamer used in the aptasensor was selected using CEM cell-based SELEX for its high specificity and affinity for a surface target. The surface target was later identified to be protein tyrosine kinase (PTK) 7.31 [33]. In this study, the sgc8c aptamers were grafted onto both the HGNs and the magnetic beads. This facilitated the selective separation of leukaemia cells from a much larger volume of the mixture sample than that used for conventional blood cell counting and separation. To further clarify the detection specificity of the HGN-based aptasensor, we performed the specificity test by detecting the same amount of control cells (10^6^ cells) and compared the signal between the target cells and control cells. As shown in Figure 5C, control cells including Jurkat, RAMOS, A549, and HepG2 cells with the same concentration were determined by the aptasensor. However, the absorbance intensity was only less than 20% of the target cells. The comparison clearly showed that the HGN-based aptasensor holds high specificity for the detection of targets.

## 4. Conclusions

In summary, we have developed a hemin-functionalized graphene nanocomposite-based aptasensor for the ultrasensitive colorimetric quantification of leukaemia cells using magnetic enrichment in this study. By simply mixing the sample with the aptamer-grafted HGNs and aptamer-functionalized magnetic beads, target cells can be captured via the specific interaction between aptamers to the corresponding targets, forming a HGN–cell–MB sandwich structure. After magnetic separation and concentration, the sandwich structures can be quantified via a colorimetric assay owing to the peroxidase activity of the HGNs. In particular, the HGNs, composed with hemins and graphene nanosheets, were employed because they simultaneously possess peroxidase-like activity attributed to hemins and the ability to absorb single-stranded DNA (aptamers) due to the presence of graphene. Furthermore, magnetic enrichment was also utilized as a signal amplification tool to concentrate cells that were of low abundance. By combining the high specificity of the sgc8c aptamer for leukaemia cells, the strong peroxidase activity of the HGNs, and magnetic amplification, an ultrasensitive assay for the CEM cells with high specificity was achieved. We demonstrated that, under optimal conditions, this aptasensor can be used to accurately determine the number of leukaemia cells to a sensitivity as low as 10 cells. We believe that the proposed biosensor holds great potential for application in a range of biomedical fields demanding more sensitive identification of cancer cells by substituting the aptamer corresponding to the cancer cells.

## Figures and Tables

**Table 1:** Comparison of analytical performance of nanocomposite-based aptasensor for optical detection of CCRF-CEM cells.

Methods/Strategy	Linear Range	Detection Limit/Sensitivity	Ref.
A label-free activatable aptamer probe-based colorimetric detection	3300–26,900 cells	3300 cells	[35]
Aptamer-conjugated polymeric (1,3-phenylenediamine resin) nanoparticles fluorescent detection	1500–3 × 10^4^ cells	44 cells mL^−1^	[36]
Graphene oxide-based aptasensor fluorescent detection	50–10^5^ cells	25 cells	[37]
Aptamer-integrated DNA nanoassembly colorimetric detection	175–1.5 × 10^4^ cells	175 cells	[38]
Multivalent duplexed-aptamer network fluorescent detection	50–10^6^ cells mL^−1^	26 cells mL^−1^	[39]
Cell-triggered cyclic strand displacement reaction chemiluminescent detection	100–5 × 10^4^ cells mL^−1^	85 cells mL^−1^	[40]
HGN-based colorimetric aptasensor	10–10^5^ cells	10 cells	This work

## Data Availability

Not applicable.

## Supplementary Materials

The following supporting information can be downloaded at https://www.mdpi.com/article/10.3390/bios12121070/s1, Figure S1: Zeta potential of Graphene, HGNs and DNA-HGNs; Figure S2: UV spectroscopy characterization and activity test of HGNs prepared with various GO-to-hemin weight ratios; Figure S3: Microscope image of capture probe-modified magnetic beads for capturing CEM cells.
