# Supplementary material for: A Hemin–Graphene Nanocomposite-Based Aptasensor for Ultrasensitive Colorimetric Quantification of Leukaemia Cells Using Magnetic Enrichment
Source: Biosensors (Basel). 2022 Nov 23;12(12):1070. doi: 10.3390/bios12121070 (PMC9776134; doi:10.3390/bios12121070)
Supplement: Supplementary file 1 [file biosensors-12-01070-s001.zip › biosensors-2005394-supplementary.pdf]

Supporting information

A Hemin–Graphene Nanocomposite-Based Aptasensor for Ultrasensitive Colorimetric Quantification of Leukaemia Cells Using Magnetic Enrichment

Jing Su [1], Liqiang Zhang [1], Luogen Lai [2], Wufu Zhu [2,*] and Chong Hu [1,*]

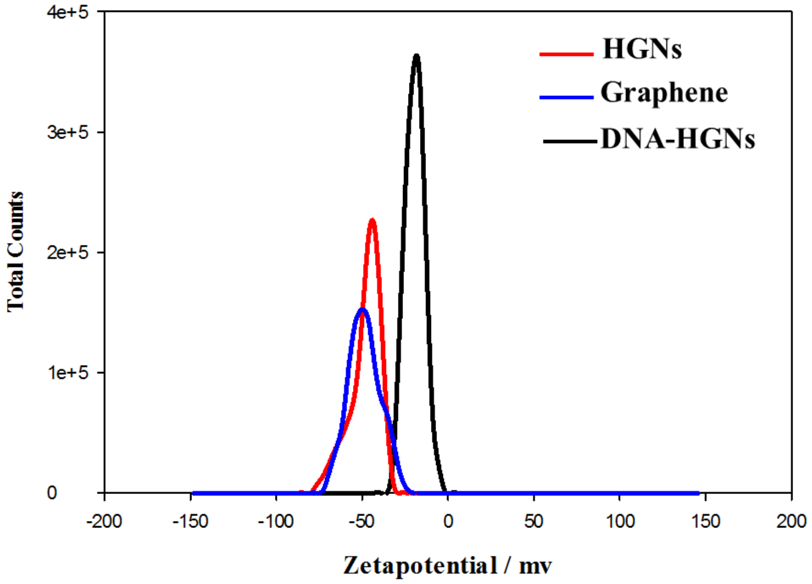

Figure S1. Zeta potential of HGNs, Graphene and DNA-HGNs.

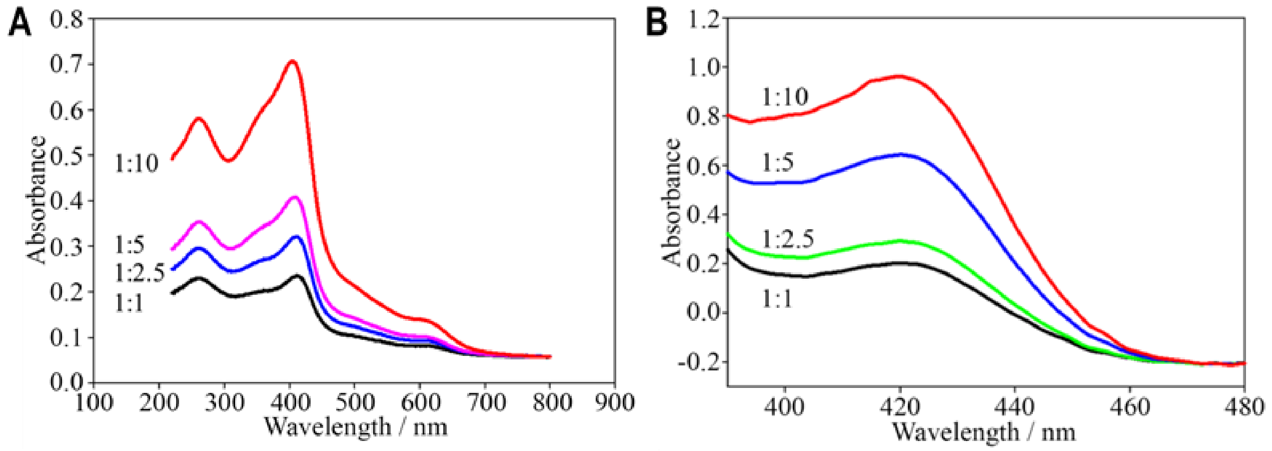

Figure S2. UV spectroscopy characterization and activity test of HGNs prepared with various weight ratios of GO to hemin. Graphene oxide/hemin ratios from 1:1 to 1:10 were tested in the preparation of HGNs, and the products were analyzed by UV spectroscopy (**A**) and for their peroxidase activities using ABTS as a substrate (**B**).

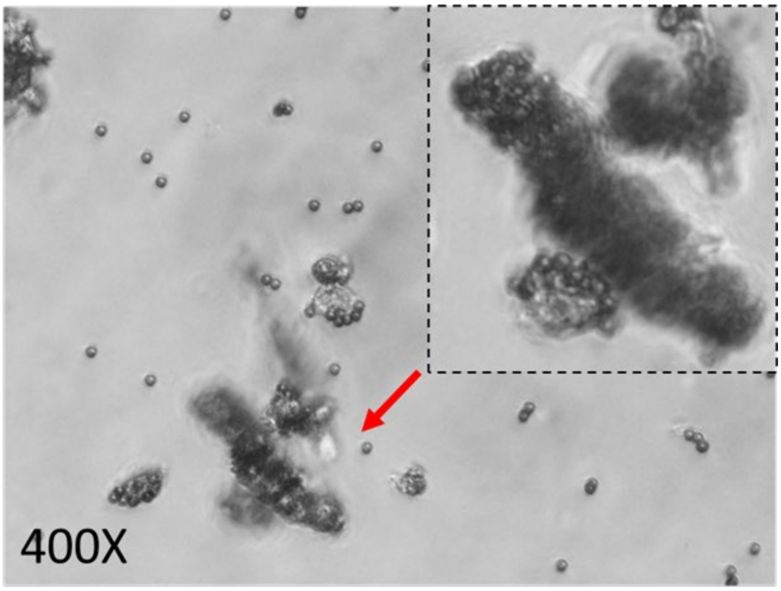

Figure S3. Microscope image of capture probes modified magnetic beads for capturing CEM cells.

$$\text{modified ratio} = \frac{A_0 - A_1}{A_0} \times 100\%$$

A_0 was the UV absorption at 260 nm before modification,
A_1 was the UV absorption at 260 nm after modification.
